# Report on Practical Medicine

**Published:** 1858-03

**Authors:** Austin Flint

**Affiliations:** Professor of Clinical Medicine and Medical Pathology in the University of Buffalo


### REPORT ON PRACTICAL MEDICINE FOR THE YEAR 1857.

By AUSTIN FLINT, M.D., Professor of Clinical Medicine and Medical Pathology in the University of Buffalo.

THE purpose of this report is to notice the more important of the contributions to practical medicine contained in the medical periodical literature of our country during the past year. Condensed abstracts of articles are given whenever practicable, and a summary of the author's conclusions when either the character or length of the papers precludes a more detailed analysis. In selecting contributions relating to practical medicine, articles treating of remedies, and, as a rule, those devoted to the consideration of the management of diseases by particular measures or methods, have been passed by. They will be embraced more appropriately in a report on materia medica and therapeutics. Contributions which relate to poisons are reserved for a report on medical jurisprudence.

In preparing this report, the various medical journals issued in different sections of the country have been examined. The reporter trusts that few, if any, important articles, within the range to which he has restricted himself, have escaped notice. He has not, however, been able to examine all the transactions of medical associations which have been published during the year, and it is a source of regret that among those which could not be seasonably procured is included the quarterly publication by the College of Physicians of Philadelphia.

The articles noticed are arranged under the following heads:—1. Fevers; 2. Diseases Affecting the Nervous System; 3. Diseases Affecting the Digestive System; 4. Diseases Affecting the Respiratory System, and 5. Miscellaneous. That other nosological divisions are not introduced, is owing simply to the fact that the important contributions to practical medicine in 1857 are found to be embraced under the foregoing heads.

The object of the report is strictly analytical. It is hardly necessary to add, that a critical examination of any of the articles noticed, or the discussion of subjects to which they relate, would have been out of place.

---

### FEVERS.

*Remitting Fever of Children.*—In a paper read at a meeting of the Society of Statistical Medicine, in the city of New York, Dr. J. LEWIS SMITH presents some considerations in reference to the nature of the disease denominated *infantile remitting fever*, and to the proper mode of treatment.

The most eminent European writers (Barthez, Rilliet, and West) describe, under this title, a form of disease which they deem identical with the continued fever of adults. Drs. Stewart and Condie, of this country, authors of works on the diseases of children, which are extensively read, regard the affection as symptomatic of intestinal irritation or inflammation. Dr. Smith has treated many cases both in private and dispensary practice, and has been led to form an opinion of the pathology of the disease differing from the views of the writers just referred to. The cases which he has studied occurred in the upper wards of the city of New York, which, as he states, are in an eminently malarious situation. He regards the disease as in a large proportion of cases miasmatic. He gives the following facts in support of this opinion:—

"1. The disease is very prevalent in spring and autumn, and rare in mid-summer and mid-winter, like malarious affections. There are certain streets where I have known it to prevail almost like an epidemic in the vernal and autumnal months. If the disease were, as Dr. Condie states, 'in every instance, either a gastro-enteritis, an ileitis, or an entero-colitis,' how can this influence of the seasons be explained?

"2. Often, not always, the remissions are more marked than would be likely to occur in a symptomatic fever. The child may appear almost well in the morning, but in the afternoon and evening exhibit such intensity of symptoms as to cause the greatest anxiety on the part of friends.

"3. The symptoms are not altogether such as we should expect to find in a purely local affection. The patient will, it is true, when asked where he feels the pain, sometimes place his hand on the abdomen, and pressure upon the abdominal parietes not unfrequently produces great distress. Dr. Condie alludes to this tenderness, evidently believing it to be a symptom of inflammation. But I have always been satisfied that it was neuralgic, from the fact that pressure on the lumbar vertebræ, and frequently on the chest and limbs, caused as much suffering as when it was made on the abdominal walls. The patient, if old enough, will complain, too, of aching in the head, back, and limbs, which is more the symptom of an independent fever than of inflammation.

"Again, constipation is ordinarily present, unless in the last stages of the disease. Intestinal irritation or inflammation, sufficient to cause so intense and protracted a fever as is often present, would be more likely to cause diarrhœa.

"4. Children, even nursing infants, take intermittent fever; why, then, may they not take remittent fever, from malaria? In my class at the dispensary, children with these diseases are frequently brought in together.

"5. I have found that measures directed to the alimentary canal, beyond simple purgation, do more harm than good. They fail to ameliorate symptoms; they awaken and distress the child. Moreover, when remissions occur, quinine will materially abridge the disease.

"6. Death seldom occurs from this affection. In one or two fatal cases which have fallen under my observation, this result followed convulsions and coma; and Dr. Stewart remarks, 'Dissections have furnished but little light on the morbid condition of the system in remittent fever; for, on a fatal termination, the transmission to the brain is the ordinary course of the disease.' The mode of death, then, and the post-mortem appearances, do not comport with the doctrine that the intestines are the seat of the morbid process.

"Dr. Condie does not agree with Dr. Stewart, but attributes death to inflammation of the intestines. I do not think that the remittent fever which I have treated, if uncomplicated, ever terminates in this way, for I have never seen a case in which abdominal symptoms did not yield to simple measures.

"7. Continued fever of the adult is of rare, and infantile remittent of frequent occurrence in the locality of my practice. The latter is not then identical with the former, as it appears to be in London and Paris, from the descriptions given by Rilliet, Barthez, and West.

"The above facts appear to me conclusive that the form of infantile remittent fever, which it has been my lot to treat, has been generally of a miasmatic character.

"It is very important to understand the nature of this affection, as the treatment will vary according to the theory we adopt. If living in a malarious region, we embrace the Broussaian views of the American authors whom I have cited, and treat the fever as a local disease, we shall fail to ameliorate the symptoms, and be mortified and discouraged by the result, if I may judge from my own experience. My reliance at present is mainly on expectant measures, till remissions occur, and then on the exhibition of quinine. In cases thus managed, convalesence has been more speedy and certain than when opium, calomel, and counter-irritation have been employed to remove intestinal irritation or inflammation.

"At the risk of appearing presumptuous, I have thus presented a theory of infantile remittent fever not, indeed, novel,—for Taylor attributes one variety of it to miasm,—but different from that contained in any American and most European treatises on diseases of children. I am the more anxious that the true nature of the disease should be understood, because I believe that the accepted doctrine is exceedingly pernicious to practitioners in malarious regions, and especially to the younger members of the profession who rely more on books than experience for guidance. The fact, too, that remittent fever has been in my practice the most frequent affection of early life, in the vernal and autumnal seasons, gives additional interest to the subject."—(*New York Journal of Medicine*, Jan. 1857, p. 106.)

---

*On the Different Methods of Treating Intermittent Fever, with the Results of Treatment in Sixty-nine Cases.* By AUSTIN W. NICHOLS, M.D., of Buffalo.

The object in this paper is to study the effect of treating cases of intermittent fever without resorting to the preparatory treatment by emetics, cathartics, etc., which are still deemed important by many practitioners; and also to institute a comparison as regards relapses and the duration of the disease, between a section of country where the disease prevails to a great extent every year, and a region not malarious.

Of the 69 cases, 46 were treated in a malarious section, and 23 in a region not malarious.

Of the 46 cases occurring in a malarious section, 17 were treated at once with quinia, in doses sufficient to arrest speedily the paroxysms, and 29 received preparatory treatment—viz. ipecacuanha and calomel, or calomel combined with either rhubarb or jalap. Relapses were observed in 14 of the latter and in 4 of the former cases, the ratio being as 1 to $4\frac{1}{2}$ in the cases which did not receive, and 1 to $2\frac{1}{14}$ in the cases which received the preparatory treatment. The average duration of the disease, dating from the commencement of the use of quinia, was found to be less in the cases which did not receive preparatory treatment, being a fraction over six days; while in the cases which received preparatory treatment, the average duration was a fraction under eight days. Adding the period occupied by the preparatory treatment, the ratio is as $6\frac{1}{3}$ to $8\frac{1}{3}$ days.

Of the 23 cases occurring in a region not malarious, all had no preparatory treatment. Of these cases, in ten previous attacks had occurred. In the latter, the average duration of the disease, after treatment was commenced, was $3\frac{1}{3}$ days. In ten of the recent cases, the average duration was $2\frac{1}{2}$ days. Of the latter, relapses were observed in two cases; of the former, in three cases.

Comparing the results as regards duration and relapses in the cases occurring in the malarious section and not receiving preparatory treatment, and in the cases occurring in the region not malarious, the contrast is striking: the average duration in the former being $6\frac{1}{3}$ days, and the average of relapses 1 in $4\frac{1}{4}$; in the latter, $2\frac{17}{19}$ days, and the average of relapses 1 in $3\frac{3}{5}$ cases. These results are greatly in favor of the region not malarious.

The reporter analyses his collection of cases with reference to the types of the disease; the number of paroxysms in each type; the number of relapses and the duration in each type. He also analyses separately the cases of tertian type.

The following summary embodies the practical conclusions which he deduces from the results of his analytical investigation:—

"From the foregoing analysis it will be seen that in cases of first attack the duration is somewhat less, and the number of cases relapsing about one-half that in cases having had one or more prior attacks. In a section of country where this fever is prevalent to a great degree every year, or in a malarious region, the duration is nearly three times that in a country not malarious, and the relapsing cases occur as frequently even under the same plan of treatment. It will be found that in a malarious region the treatment of patients by quinia alone not only diminishes the number of paroxysms and abridges the duration of the disease, but that fewer relapsing cases occur than where a preparatory course of treatment has been adopted. Even in the analysis of 40 tertian cases, although the duration of the number of paroxysms are nearly the same under the two different methods of treatment employed, yet the cases of relapse are found to be *nearly twice* as numerous where the preparatory plan was adopted."—(*Buffalo Medical Journal and Monthly Review of Medical and Surgical Science*, January, p. 460.)

---

*Treatment of Remitting Fever.* By JNO. HERBERT CLAIRBONE, A.M., M.D., Petersburg, Virginia.

The practice of Dr. Clairbone, in cases of remitting fever, is embraced in the following quotations from an article entitled "Periodic *vs.* Typhoid Fever."

"It is in the treatment of these cases the 'triple base' of Maillot so accurately expresses the indications to be fulfilled—viz. 'To combat the visceral lesions; to oppose the return of the paroxysms; to prevent the occurrence of relapses.' To carry out the first, it may be only necessary, if the patient be seen in the paroxysm, to administer six or eight grains of the mild chloride of mercury, with as many of Dover's powder, and one or two of ipecac., applying a dozen or two leeches to the head or stomach, according to the force of the reaction and its concentration at either point. The early occurrence of the remission will afford opportunity to exhibit the anti-periodic, which will effectually meet the two latter. Fifteen or twenty grains of the sulphate of quinine given in one dose at this time, or in two doses of a few hours' interval, will usually cut short an attack. Indeed, I have seen it succeed, in summarily effecting this end, after the disease had already continued unabated for more than a week, and when a dry tongue, nervous tremors, and incoherency of language had apparently ushered in the typhoid stage. After two or three days of treatment, if the fever still continue, which is sometimes the case, we have found smaller doses of quinine, five or six grains, exhibited in the remission, to answer a very good effect—gradually neutralizing the poison of the disease, and hastening convalescence, without inducing any of the disagreeable symptoms of cinchonism. We sometimes combine the quinine with calomel, ipecac., and opium, at its first administration; and where there is much visceral engorgement, the antiperiodic is often thus more effectual.

"Cases subjected to this treatment in their early stages have not generally, in our experience, 'run into typhoid fever.' Of nineteen cases, not selected, but transcribed from our note-book of last September, the average duration of treatment was six days. Six more days would cover the average period of convalescence. * * * * *

"When the fever has persisted for one or two weeks, in spite of the treatment adopted, and the tongue begins to be dry, and brown, and fissured, and the bowels are irritable, we usually recommend, about once in twenty-four hours, four or five grains of Dover's powder, with as much of hyd. c. creta, if there should be a necessity for the latter in the condition of the secretions, and apply at the same time a mild vesicatory over the abdomen. We continue the use of the antiperiodic, however, exhibiting three grains of quinine or an ounce of the infusion of cinchona and serpentaria every six hours, alternating sometimes with fifteen or twenty drops of oil of turpentine.

"With regard to the use of purgatives. We have found them generally not only unnecessary, but positively prejudicial at any stage of the disease, and evincing, even the mildest of them, aptness to induce irritation of the bowels."

---

*Treatment of Scarlatina.* By JAMES D. HARPER, M.D., Minden, Louisiana.

After describing briefly the characters of an epidemic of scarlatina which prevailed in Minden in May, 1856, Dr. Harper gives the following account of his method of treatment, and the results.

"Of the 40 cases under my care during the epidemic, there were 19 of simplex, 12 of mild anginosa, and 9 aggravated cases of anginosa, some of which threatened to assume the malignant form. In some of the cases, seldom anything was employed beyond a saline laxative, repeated from time to time, as circumstances demanded. When the arterial excitement was very high, *cold water to the head and throat,* cooling drinks, and gargles of flaxseed and vinegar, constituted the treatment in a majority of the simple cases; in others, the chlorate of potash was added to the treatment, as recommended by Dr. Watson, with the happiest effect. The twelve mild cases of anginosa yielded pretty much to the same treatment as those of the simple form,—the more lavish use of *cold water,* and the occasional application of nitrate of silver or sulphate of copper, with the probang, constituting the essential difference in the treatment of these two forms of scarlatina. The troublesome itching of the skin, which is apt to supervene upon the appearance of the eruption, can be relieved to some extent by sponging the surface with cold water, which does away with the intolerable heat; brans of different kinds may be rubbed upon the body, and thrown into the bed of the patient, which soothes for a time.

"The nine cases, alarming in their character, underwent in the early stages, to a certain extent, the same treatment as those of the mild form; but as they advanced, the more threatening become the symptoms, such as great difficulty of breathing, from glandular enlargement, combined with a viscid and tenacious secretion, choking up the larynx; a more frequent pulse; extreme restlessness and jactation, doubtless arising from obstruction in the air-passages, with some disposition to diarrhœa, and evident tendency to congestion of the brain; in some, these symptoms were recognized at an early period; in others, were deferred until desquamation had commenced. The head and throat trouble was the distinguishing feature in these cases; pulse ranged from 125 to 180, with more than usual heat about the head, attended at times also by slight incoherency, and nervous twitchings of the fingers and eyelids. There is but little doubt that in most of these cases the excitement of the brain evidently arose from the condition of the throat; these symptoms were, however, promptly met, not by sponging, but by *pouring cold water* out of a pitcher, or some other convenient vessel, upon the head, giving the water a fall of from 4 to 12 inches; this was continued from 15 to 40 minutes at a time, or until the pulse was reduced 20 or 30 beats in a minute, and the head became cool. At this crisis, the patient would more frequently than otherwise go off in a refreshing sleep, lasting half an hour, more or less. Reaction manifested itself by the same restlessness, incoherency, and hurried respiration, but which would always give way to the impression of the water, thereby placing the patient in a condition highly favorable for recovery, other things being equal. In some of the cases *chlorine* was used as a stimulant and antiseptic with good effect.

"There was a condition of the throat which gave rise to much trouble, even after desquamation had in some instances almost subsided, and the patient otherwise convalescing; it was where the ulcer was so low as to be beyond the reach of the probang, and the little patient, not knowing the importance of raising the foul secretions, swallowed them, whereby the system became thoroughly inoculated with the animal poison, generating that condition of system well known as typhoid, and in one instance producing, as I am well convinced, that characteristic feature of the diseased state of the glands of Peyer, similar to that which accompanies genuine enteric fever, with a pulse from 125 to 180, a tympanitic abdomen, and considerable excitement about the brain. Cases of this character were treated pretty much as those of enteric fever, at the stage where similar symptoms were present, with the addition of cauterizing the ulcer of the throat, if accessible, which is most certainly the source of the mischief. A nourishing diet was given, also brandy or port wine, with a free use of chlorine, to rid, as much as possible, the secretions of their obnoxious properties. When the respiration became embarrassed or hurried, from flatulency, turpentine was administered; if any threatening of brain disease appeared, the patient was subjected to the cold water, and continued until such symptoms were allayed.

"This condition of things lasted from six to ten days. The motions from the bowels consisted mostly of secretions from the throat, occasionally tinged with fæcal matter; but so long as the throat remained in an ulcerated state, diarrhœa was invariably present; it could only be temporarily checked, as the source of the malady had to be reached before any permanent benefit could be realized, proving that the diseased throat and enteric symptoms bore the relation of cause and effect.

"The sequelæ of 'scarlet fever' are generally a source of much interest and uneasiness to the physician. Indeed, there is no one disease which presents a longer catalogue of secondary affections than scarlatina. Of the various diseases which are likely to follow scarlet fever, dropsy in some form is, according to my observation, the most frequent. Of the various forms of dropsy, anasarca is probably the one generally met with; it ordinarily makes its appearance from the last stages of desquamation to the third or fourth week following. Medical opinion is yet unsettled as to the form of scarlatina most apt to be succeeded by dropsy. Of the fifty-six cases enumerated, there were eight cases of anasarca, all of which supervened upon mild cases of scarlet fever, not one severe case of scarlatina resulting in dropsy of any kind. In the dropsies following this eruptive fever there is an almost total inaction of the kidneys, an inertness which seldom attends the disease originating from a different cause; it readily yields to active hydragogue cathartics and diuretics—the one to produce copious watery evacuations from the bowels, while the other incites the kidneys to increased action. An exclusive milk diet should be rigidly adhered to throughout the complaint. When the lower extremities are swollen to any great extent, a bandage applied, beginning at the toes and terminating at the knee, or high up the thigh, if preferred, has been found highly useful in reducing the effusion. After the effusion has been in a great measure removed, if the patient is perceived to be in an anæmic condition, some one of the mineral acids, or ferruginous preparations, should be immediately resorted to. A varied and nutritious diet, in this state of the system, is admissible.

"It is stated that the dropsies which follow scarlatina are sometimes dependent upon, or associated with, that peculiar uræmic state known as 'Bright's Disease;' if so, then a case thus dependent on, or associated with 'Bright's Disease,' would be found more difficult of cure than those which came under my notice.

"Various speculations have been indulged in regard to the prophylactic virtues of atropa belladonna in scarlatina. There are few who place implicit reliance in its preventive properties; others, again, firmly believe that it has the unmistakable power of *modifying*, if not preventing, an attack; while others, whose statements are equally reliable, denounce its virtue in preventing or modifying the disease, and, in this respect, as worthy only of the source from which it emanated.

"I gave belladonna, during the epidemic of 1856, to 20 children; 19 of them took it as directed, and one irregularly. Of the 20 children, the one only who neglected to take the medicine as directed had the disease; most of the 19 who escaped were not only exposed to the epidemic influence, but to direct contagion.

"With this experience, I have ranked myself with that class of the profession who, without relying implicitly on the preventive powers of belladonna, yet deem it highly useful in modifying and arresting, to some extent, the dreadful ravages of scarlatina."—(*New Orleans Medical and Surgical Journal*, May, 1857, pp. 743–746.)*

---

*Hospital Treatment of Yellow Fever in New Orleans.* By R. D. POWELL, M.D., Brunswick Co., Va.

Dr. Powell states that, having been a resident of the Charity Hospital in New Orleans in 1855, he had an opportunity of comparing different methods of treating yellow fever, and the plan which appeared to him most successful was the following:—A hot mustard foot-bath was promptly employed. Next, the infusion of orange-leaves given freely as a drink, and continued during the attack. As a purgative, castor oil combined with a few drops of laudanum. This was administered soon after the admission of the patient. Ice applied to the head to relieve cerebral symptoms, if the latter supervened; also, local blood-letting, by cupping or leeching.—(*Virginia Medical Journal*, June, 1857, p. 470.)

---

*Epidemic Fever characterized by mild Erythematic Pharyngitis.*

During the months of January, February, and March, 1857, there prevailed, in the city of Buffalo and its vicinity, a form of fever accompanied by mild pharyngitis, having a career of from three to five days, and generally, if not invariably, ending in convalescence. In a report made to the Buffalo Medical Association by the reviewer, the results of an analysis of twenty-three recorded cases coming under his own observation were given, and the question of the identity of the disease with scarlatina discussed. From the results of the analysis, the following deductions were drawn by the reporter:—"The disease was an epidemic fever, characterized by mild erythematic inflammation of the fauces as a constant local complication. Its character as essentially a fever is established by the febrile movement being in so marked a degree out of proportion to the local affection; in other words, evidently not being symtomatic of the latter, and by its running a definite although a brief career. It is a fever of from three to seven days' duration. Its epidemic character is sufficiently

---

* An interesting article by Dr. Lutton, of Aurora, Indiana, on the diversity of symptoms in scarlatina maligna having already appeared in the pages of this journal, (North American Medico-Chirurg. Review,) No. for Nov., p. 912, is not here introduced.

apparent. It has prevailed more or less extensively in the city for about two months, reaching its acme gradually, declining gradually, and at length disappearing, affecting both sexes and different ages without notable discrimination. As an epidemic fever its symptomatic features were very uniform. The erythematous affection of the fauces constitutes the only positive character, aside from the brief duration of the febrile career. The other symptoms uniformly present were only those incident to febrile movement; and the symptoms observed in a few cases—viz. the convulsions in one case, the retraction of the head in one case, etc., were only incidental events, not intrinsic elements of the disease. The small patches of white exudation observed in some of the cases do not suffice to establish any relation of the local affection to that called *diphtheritic* by Brettonneau and others. The occurrence of several cases repeatedly in the same family does not suffice to prove that the disease was propagated by contagion, since this fact is explicable on the supposition of the patients being equally exposed to an epidemic influence, and there being a marked discrepancy in the intervals separating the cases necessarily occurring in the same family.

The disease was considered as a species of fever distinct from scarlatina, on the following grounds: 1. The uniform absence of the scarlatinous eruption on the exterior surface. 2. The uniform absence of any connection with well-marked cases of scarlatina, occurring either previously or subsequently. In no instance was the disease preceded or followed by scarlatina in the same family. 3. Several of the persons affected being adults and persons beyond middle life. 4. Medicine was in no instance followed by the sequels of scarlatina—viz. rheumatism, serous inflammation, and especially dropsy. 5. In several instances the persons affected had had scarlatina.

Professor Rochester at the same time made a report on the same subject. Between January 6th and April 4th he had noted thirty-seven cases. In many instances he had been led to observe irregular intermittency in the febrile movement. The subjects in ten cases were of all ages and conditions. He regards the communicability of the disease as probable. If communicable, the period of incubation is short, the disease manifesting itself in some instances within twenty-four hours after exposure. In many of his cases the patients had had scarlatina. Two children who were very ill with scarlatina had this form of fever a month after their recovery.—(*Buffalo Medical Journal*, May, p. 718.)

---

### DISEASES AFFECTING THE NERVOUS SYSTEM.

*Hydrophobic Hysteria.* By Prof. D. L. McGugin, A.M., M.D., of Keokuk, Iowa.

Professor McGugin reports a case of hysteria, in which the symptomatic phenomena characteristic of hydrophobia were exhibited. The patient was a young married female of extraordinary mental acquirements, and highly impressible. The attack occurred shortly after the death of an infant, her only child. It commenced with pain in the head, without febrile movement. The bowels were constipated, the urine pale, and an attempt at swallowing was undertaken with reluctance and performed with difficulty. Shortly after, she became subject to paroxysms, during which she complained of thirst and demanded water, but on bringing it to her lips violent spasm of the larynx was excited. "When first attempting to drink, the appearance of the water seemed to produce a slight shivering, as if of dread, and after some hesitation she would hurriedly, apparently with a feeling of desperation, seize the cup and with a sudden effort attempt to gulp it down, during which respiration was suspended; and after the effort at swallowing, her conduct resembled that of an individual who had been for a time immersed in water of a low temperature. Then followed a wild expiratory screech. During these fits her countenance manifested the wildest frenzy, and all the muscles of the body seemed tensely contracted." These paroxysms were excited by the unexpected entrance of strangers, a sudden noise, or a current of cold air.

Aphrodisiac symptoms supervened, and she finally fell into fatal coma. The case was treated by antispasmodics and the inhalation of chloroform. It is reported with reference to the simulated phenomena of hydrophobia.—(*Iowa Medical Journal*, July and August, p. 103.)

---

*Epilepsy treated by Ligation of the Common Carotid Artery.* By C. ANGELL, M.D., of Pittsburg, Indiana.

Dr. Angell has resorted to ligation of the common carotid artery on one side in two cases of epilepsy. The first operation was performed in July, 1857. The patient, a male, was twenty years of age, about five feet in height, large head, short neck, sanguine temperament, and of full habit. Epilepsy had existed for three or four years, the fits progressively becoming more frequent and severe. He had become partially idiotic. He had from fifteen to twenty fits during the forenoon of the day on which the operation was performed. The day after the operation he complained of difficulty in swallowing, and the left side became incompletely paralyzed. The paralysis continued, with difficulty of articulation and swallowing, till the next day, when he died in a comatose condition. The epileptic paroxysms did not recur after the operation.

The second operation occurred a few days after the first. The patient, a male, was forty years of age, of a full habit and sanguine temperament. Epilepsy had existed for seven years. For the last three years the paroxysms had recurred almost daily. The mind was much affected. He recovered from the operation satisfactorily, and had no return of the epilepsy for twenty-two days. At the end of that time he had a paroxysm on two successive days. Seventeen days after this a third paroxysm occurred, and about a month afterward a fourth. These four paroxysms were all that had taken place up to the time of writing the report, a period of a little more than two months. The paroxysms after the operation differed from those which occurred previously as regards premonitions. Prior to the operation, he had no warning of their approach; but after the operation, a sensation of dizziness preceded the attacks for several minutes, giving him time to provide against falling.

The patient declared that he felt better than at any time during the three years; some of his friends thought that there was a decided improvement in his general appearance and mental condition.

The report is made too recently after the operation to warrant any conclusion as to its permanent value in this instance.—(*North-Western Medical and Surgical Journal*, October, p. 446.)

---

*To what Degree are the Intellectual Faculties affected in cases of Apoplexy and Hemiplegia?* By BENJAMIN W. M'CREEDY, M.D., Physician to Bellevue Hospital.

This inquiry, irrespective of its medico-legal relations, is of interest and importance to the medical practitioner. Dr. M'Creedy brings to bear upon it the results of an analysis of cases collected from different authors, as well as instances falling under his own observation, and communicated to him by medical friends. From an examination of "a collection of cases of apoplexy," by Mr. Copeman, of London, published in 1845, he arrived at the following conclusions: "In all, out of a record of two hundred and fifty cases, fifty cases have been taken in which the patient had recovered from the first effects produced by the apoplectic stroke. They are all the cases contained in the book in which such recovery had taken place, and in which the cases were clearly of an apoplectic character. Yet in no one of the cases is there the slightest indication that the patient was left with a mind, I will not say reduced to a state of imbecility, but impaired in any marked degree. They all, as far as the mind is concerned, with the exceptions to be mentioned, recovered; recovered perfectly; were restored to their usual state; returned to their previous occupations. In two instances the faculty of speech was either deranged or lost, and in those instances their physicians assert that the faculty of language, not reason, was deranged. In the third instance the patient is stated to have recovered with an impaired memory, and with some confusion of thought; but a merchant, seventy-three years of age, who, after partial recovery from a severe apoplectic seizure, is enabled to labor for his support by writing for the weekly papers, may be esteemed to have still possessed a fair share of intellect and energy. The second attack left him yet more prostrate, very much weakened in body, and unable to do anything for the support of his family. From the third attack he never recovered; still there is nothing like imbecility: he lies for months in a feverish condition, alternating between coma and delirium, and even a few days before his death he partially recovered and talked in a rational manner with his wife."

The foregoing conclusions are corroborated by the results of an examination of a series of cases contained in Andral's *Clinique Médicale*, and in the *Anatomie Pathologique* of Cruveilhier. These cases, like the preceding, are quoted in brief detail by the writer. In expressing the results of this examination, the writer says: "Here, then, are sixteen cases in which the condition of the intellect before the attack was obscured, in which the nature of the disease was verified by post-mortem examination—being all that are contained in the *Clinique Médicale* of Andral, and the *Anatomie Pathologique* of Cruveilhier, and reported by men whom every one will allow were competent, careful, and conscientious observers—in which the hemorrhage was situated in almost every part of the brain, presenting every degree of severity compatible with the continuance of life, many of them living for years after the occurrence of the apoplectic seizure, and yet in only two of these was there any decided improvement of the mind noticed."

The writer adds: "Perfectly in accordance with this have been the results of my own observations. At Bellevue, the great pauper hospital of the city, there are always a number of hemiplegiacs; but since my attention has been directed to the subject, I have seen no case in which hemiplegia has been the consequence of a well-marked apoplectic seizure, in which I have found the intellect seriously impaired. In some of these cases the first impression of the observer is wholly unfavorable to the intelligence of the patients; the distorted countenance, the impaired speech, and the motiveless tears or laughter, give them an appearance of utter imbecility; yet a patient examination will commonly discover an amount of intelligence entirely unexpected." * * * * "From the facts given above, no other conclusions can be drawn than that any impairment of mind, as a direct consequence of apoplexy, after the patient has recovered from its primary effects, must be an exceptional occurrence. That the apoplectic seizure may hasten the approach of senile atrophy of the brain is, as before stated, probable; when atrophy has already commenced, an apoplectic attack may undoubtedly quicken its progress, and, in such a case, the friends of the patient would naturally attribute the rapid decay of the mind wholly to the apoplectic seizure. This, I think, I have myself seen; and as apoplexy becomes more common as life advances, such cases may not be unfrequent. The confusion of mind, the difficulty in pursuing a train of thought, of which apoplectics are apt to complain, is to a great extent the mere result of diminished nervous energy. They comprehend well, and judge correctly, but, before their general health is confirmed, they can no more think continuously than they can take a long walk, or perform any other act demanding a considerable expenditure of nervous force. It is not the brain specially that is affected—it is the system at large. Of all the faculties, memory, either special or general, is most apt to be impaired, and this impairment patients are always ready to admit and complain of. As the patient recovers, the memory commonly improves; and if no new attack supervene, this improvement is progressive for years." * * * "Whence, then, arises the number of imbeciles who are to be found in every almshouse? Partly, doubtless, from the cases already mentioned, in which senile atrophy is prematurely caused, or its progress hastened by the occurrence of apoplexy. And let us recollect the state of isolation and neglect from which such persons frequently, often necessarily suffer, is in itself a main cause of a weakened intellect: the mind rusts out for want of exercise."

The writer details a series of cases in which, while the hearing remained unaffected, the patient's consciousness entire, and no delirium, the faculty of speech was either lost or perverted. He remarks on these cases as follows:—"Here, it will be seen, are eleven well-marked cases of hemiplegia, three of them complicated with epileptic convulsions, and all of them with loss or perversion of the faculty of speech. Besides the cases here recorded, three more have come to my knowledge the subjects of which, I believe, are still living. This shows conclusively the loss of speech, as an accompaniment of hemiplegia, to be no *very* rare occurrence. Of the whole fourteen cases, two have perfectly recovered the use of speech; two have recovered it to a partial and limited extent; the others still, I believe, remain speechless. From the cases recorded in the journals to which I have access, I believe that recovery from perversion of the faculty of speech is more common and perfect than when the power of articulation is either wholly lost or confined to a few monosyllables. Direct medication, except in so far as it improves the general health of the patient, seems to have no effect on the lost faculty; more is to be hoped for from careful, long continued, and well-directed exercise of the local organs by the patient himself."

The foregoing conclusions are certainly at variance with the views inculcated by medical writers and commonly held by practitioners. The general belief is, that after an apoplectic seizure, as a rule, the mind is more or less impaired, and in severe cases the patient is apt to fall into a condition of partial or complete imbecility. Epilepsy is also supposed to tend intrinsically to induce deterioration of the mental faculties in proportion to the frequency and severity of the paroxysms. Assuming that the writer's conclusions are correct, it is in a great measure owing to the prevailing error on the subject that the faculties of the mind are, in a certain proportion of instances, permanently damaged; and it becomes a very important point in practice to enjoin, under judicious restrictions, that degree of exercise of the mental faculties which shall secure their healthful if not vigorous activity. This practical point is especially applicable to the management in cases of epilepsy. The reviewer has long entertained the opinion that, in this affection, the mental deterioration which is frequently observed is not due so much to an intrinsic tendency of the malady as to the isolation and inactivity incident to it. With reference to epilepsy, the author of the highly interesting paper which we have reviewed, states that he has "repeatedly seen and prescribed for a person who has had epilepsy for *thirty-three years,* occurring several times in every month, and sometimes eight and ten times a day; in whom, though there is obvious enlargement of one side of the head, and much headache, and the tongue bears the marks of countless lacerations, yet it is impossible to discover in this person the least change in her mental condition." He adds, that "both the medical profession and the judicial bench of this city can each furnish at least one illustration of the distinction and usefulness to which persons who have been, or still are, subject to epilepsy, may attain."—(*New York Journal of Medicine,* September, p. 203.)

---

*Case of Ecstasy;* reported by A. W. NICHOLS, M.D.

This singular and rare neuropathic disorder was exemplified in the case of a German, a mechanic, aged twenty-five years, admitted, August 1st, into the male ward of the Buffalo Hospital of the Sisters of Charity, under the charge of Dr. Flint. He was apparently in a state of insensibility when admitted, and could not be roused to any manifestation of consciousness. On raising the eyelids they remained open for some time, and he apparently took no notice. Flies creeping on the face, and even on the conjunctiva, did not disturb him. The respiration was perfectly normal; the pulse 76 and regular; no distension nor tenderness of abdomen. Drinks and nutriment introduced into the mouth were retained for some time and mostly escaped, a small portion being swallowed.

He lay motionless, not changing his position nor giving any indication of suffering. A bottle of ammonia applied to the nostrils occasioned contraction of the orbicular muscle of the mouth and flushing of the face, but no other manifestation of discomfort, nor efforts to escape from the inhalation. His countenance denoted quiet sleep.

He remained in this condition until August 4th. At the end of this period the only change consisted in his keeping the eyes opened a part of the time, apparently taking no notice, the eyes fixed in one direction, winking but seldom; and in his voluntarily altering his position from lying on the back to lying on the side. The catheter was introduced without resistance on his part, or difficulty, and a quart of urine removed. The iris, all along, was sufficiently mobile. The only treatment pursued up to this time consisted of enemas of spirits of turpentine and the tincture of assafœtida, with the forcible administration of brandy and nourishment.

On the 5th inst. there was marked improvement. He lay with his eyes open, and the countenance indicated more intelligence. He made slight, ineffectual efforts to protrude the tongue. He had asked for drink; complained of inability to hear, and for a time wept violently.

On the 6th his condition was not so good; he resisted taking food; lay with eyes open, but offered to take no notice. He passed urine in bed. No dejection since his admission.

He remained without any material change till the 8th inst. Firing, with a hammer heated by boiling water, was resorted to. This was borne for some time, but on continuing the application he at length began to make vigorous resistance, and finally drank brandy and water freely. He could not be made to speak.

On the 10th he took drinks and nourishment freely, and replied to questions. The previous day the direction had been given at the bed-side to resort to severe firing if the speech were not recovered.

On the 12th the patient was taken from the bed and seated in a chair. He remained passive, and his expression denoted great dejection. He did not reply to questions, and nourishment was given with considerable difficulty.

On the 13th he was carried into the open air. On the 14th there was a marked change. His expression was entirely altered; he took notice, smiled, and offered his hand. Walked of his own accord, with a little aid.

On the 15th he relapsed into his former condition, and firing was resorted to. He remained in this condition till the 18th. Firing and the cold douche were employed. By these means he was made to take nourishment. By the same means he was at length made to groan, to speak, and get up. For several days his countenance had an air of extreme abstraction. The bowels remained constipated.

On the 26th of August he walked about, ate freely, and answered questions readily, but talked as if the mind acted slowly. He was still disposed to keep his eyes fixed on one object, and, except when addressed, had a contemplative aspect. He gradually improved, and left the hospital reporting quite well, September 23d.

It was impossible to obtain definite information respecting the state of his mind before and during the state of ecstasy. There was no evidence that the attack was due to religious excitement. A similar attack had occurred several months before.—(*Buffalo Medical Journal*, August, p. 139.)

---

*Case of Trismus Nascentium, ending in recovery.* By H. L. BYRD, M.D., Professor of Principles and Practice of Medicine, in Oglethorpe Medical College.

Professor Byrd states that, in a practice of sixteen years, he has seen over thirty cases of trismus nascentium, all of which terminated fatally, with the exception of the single case now reported. The child, a negro, was attacked eight days after birth. The case presented no unusual features. The treatment consisted of flaxseed poultices over the abdomen; enema, containing a drachm of castor oil and ten drops of the spirits of turpentine, and five drops of spirits of turpentine and paregoric, given by the mouth every two hours. The latter was given irregularly, owing to the spasms. On the fourth day, the symptoms being improved, one-fourth of a grain of the sulphate of quinia was given every three hours, alternating with eight drops of the spirits of turpentine. The reporter attributes the recovery mainly to the spirits of turpentine.—(*Charleston Medical Journal and Review*, July, p. 474.)

---

*Recovery from Paralysis.*—Paralysis of considerable duration, it is well known, may continue indefinitely after the primary condition inducing it is removed, and when the nervous and muscular systems have not suffered lesions which are incompatible with the recovery of the power of voluntary motion. The paralyzed muscles do not, of their own accord, return to the dominion of the will, but efforts directed by the physician to this end are indispensable to recovery. This practical point is not always sufficiently considered by practitioners, and many persons remain paralytics in default of proper measures to effect recovery. This fact is illustrated by the reported success of gymnastic exercises in the hands of Dr. J. P. Batchelder, of New York. By a system of muscular training, patiently pursued for several months, he has succeeded in accomplishing surprising results in cases of paralysis of several months' standing.—(*Editorial Correspondence in Medical and Surgical Reporter, Burlington, N. J.*, July, p. 356.)

---

*Intercostal Neuralgia.* By A. W. NICHOLS, M.D.

Dr. Nichols treats of the causes, symptoms, diagnosis, and treatment of intercostal neuralgia, his remarks being based on an analysis of twenty recorded cases. The results of the analysis go to show that this affection generally occurs after puberty, and under thirty-five years of age. It occurs in persons not enfeebled, but apparently possessing good constitutions. It appears to be developed in those who are engaged in active occupations rather than in those of sedentary habits. All but two of the cases analysed were observed in the latter part of winter and early part of spring. The left side is more commonly affected than the right. Both sides are rarely affected. It is sometimes observed in the course of pulmonary tuberculosis, and frequently during or after an attack of periodical fever.

As regards the symptomatic phenomena, the histories of these cases confirm the correctness of the descriptions of the affection given by Nicol, in 1818, and, more recently, by Bossereau and Valleix. The most distinctive feature is the existence of pain, and tenderness on pressure, limited to particular points on the thoracic and abdominal parietes. These points are, *first*, slightly to the outside of the spinous processes of the dorsal vertebræ and almost over the dorsal-intervertebral fosamina, just opposite the divisions of the spinal nerves into their anterior and posterior branches; *second*, about midway between the spinous processes and the margin of the sternum; and, *third*, near the edge of the sternum, or on the abdomen at the side of the median line. The pain or tenderness may be limited to one or two of these points, and often all three are affected. The tenderness is very circumscribed, extending over a space which may be covered with the ball of the thumb.

The treatment which seemed most efficient consisted of vesication, anodynes, and tonic remedies. The affection was found to persist, in several instances, for some weeks.—(*Buffalo Medical Journal*, July, p. 65.)

---

*Cases of Partio-General Paralysis, or the Paralysis of the Insane.* By PLINY EARLE, M.D.

Dr. Earle proposes the above title for the affection, first described minutely by M. Calmeil, of Paris, called by the French writers *Paralysie général progressive*, or *Paralysie des Aliénés*, and by the English and Americans, *Paralysis of the Insane.* He reports five cases. The morbid appearances after death in these cases are given. One of the cases is remarkable from the fact of recovery having taken place. Dr. Earle states that this is the only instance of recovery that has come under his observation; and that M. Calmeil, who has been connected with the hospital for the insane at Charenton, Paris, for twenty years, has never known a case of complete recovery. The cases are reported in full detail, and do not admit of being condensed.—(*American Journal of the Medical Sciences*, July, p. 36.)

---

### DISEASES AFFECTING THE DIGESTIVE SYSTEM.

*Cholera Infantum.* By EDWARD H. PARKER, M.D.

In a paper read before the New York State Society, February 4th, 1857, Dr. Parker discusses the following propositions, as those generally held by the profession, or, at least, as generally given by our systematic writers :—

1st. "Cholera Infantum is a disease peculiar to this country, though not entirely unknown in Europe.

2d. "That Cholera Infantum is almost entirely confined to large cities, and is rarely seen in the country.

3d. "That the symptoms, course, and pathology of the disease entitle it to a separate place in our nosological tables.

4th. "That the treatment is to be distinct from that of diarrhœa, on the one hand, and dysentery on the other.

5th. "That perhaps the most alarming symptoms are those of hydrocephalus, occurring in the advanced stages."

With reference to the *first* proposition, he quotes descriptions by Dr. West, of London, of an affection called *inflammatory diarrhœa*, and by M. Bouchut, of Paris, *entero-colitis*, either of which would answer for a description of the cholera infantum of this country.

The *second* proposition he deems incorrect, and adduces facts obtained from the mortuary statistics of the last century to show that the affection is as fatal in the country as in the city.

As regards the *third* proposition, the writer arrives at the conclusion that it is impossible to draw a line, on the one hand, between cholera infantum and simple diarrhœa, and, on the other hand, between cholera infantum and entero-colitis. He claims, therefore, that cholera infantum is, in fact, a name given to a particular condition arising in other diseases.

The foregoing conclusion respecting the *third* proposition involves a denial of the *fourth;* for if there are no peculiar symptoms, course, and pathology, there is no separate and peculiar treatment.

In commenting on the *fifth* proposition, the writer calls attention to the distinction between the *hydrocephaloid* and the *hydrocephalic* condition; the former, as described years ago by Marshall Hall, and more recently by Gooch and West, being the condition occurring in the course of cholera infantum, and demanding the treatment due to anæmia and exhaustion, and not to encephalic inflammation.

In conclusion, the writer presents the following propositions as expressing his views, in contrast to the propositions already given as expressive of the opinions more commonly entertained.

"1. The condition to which the name of cholera infantum is given is not a separate and peculiar disease, but a collection of symptoms attendant on certain stages of other diseases.

"2. This condition is recognized by European authors in their treatises, though very properly it does not receive a separate title.

"3. That the principles of its treatment are the same as those of diarrhœa and entero-colitis.

"4. That hydrocephalus is rarely, if ever, an attendant or sequent of it, but that the hydrocephaloid disease is very usual."—(*American Medical Monthly*, May, p. 257.)

---

*Epidemic Dysentery, as it Prevailed in the Sequatchee Valley in* 1856. By A. FRAZIER, M.D., Pikeville, Tenn.

In the situation above mentioned, within a range of ten or fifteen miles down the valley, there occurred, during the summer and autumn of 1856, about sixty deaths from epidemic dysentery, in a population of not over two thousand. The epidemic was characterized by typhoid symptoms, which were often present from the commencement. Dr. Frazier argues that in an epidemic form the disease is something more than an inflammation of the mucous membrane of the intestine; that it involves a morbid condition affecting the entire system, of which the intestinal inflammation is the local expression. He opposes the notion, still adhered to by some practitioners, that the pathology of the disease consists in congestion of the portal circle proceeding from hepatic obstruction. His experience leads him to reject the free administration of calomel, especially in epidemic dysentery with an adynamic tendency. Stimulants and sustaining diet he regards as highly important in such cases.—(*Southern Journal of Medical and Physical Sciences*, April, p. 231.)

---

*Dysentery and its Treatment.* By HENRY TIEDEMAN, M.D., Philadelphia, 1857.

In a pamphlet of twenty-four pages the author defines, as far as the limited space admits, his views of the nature of dysentery. He attributes dysentery to inflammation of the sub-mucous areolar tissue of the rectum, extending to the mucous membrane. This inflammation is caused by disturbed circulation of the blood in the liver. The author rejects miasma as a cause of dysentery, and denies that dysentery is a malarious disease, or has any affinity to intermittent fever, or has anything to do with a virosus or toxicosis.

The first symptoms of dysentery are caused by hyperæmia in the capillary system of the rectum, in consequence of repletion in the large veins of the liver and disturbance of circulation in those vessels. The author draws a parallel between hemorrhoids and dysentery, and adds, that he never found a patient afflicted with hemorrhoids who was attacked by dysentery, unless the former disease had been interfered with by improper treatment.

According to Dr. Tiedeman there are only two reliable symptoms of dysentery—viz. tenesmus and the dysenteric sedes. All the generally adopted different species of dysentery are rejected as mere complications or modifications of the disease.

The treatment is very simple, and therefore would be highly recommended. The author states that of a large number of patients which he has treated during the last six years, none died. The treatment employed is only applicable in the first two stages. The patient very seldom reached the second, never the third stage of the disease. There were but few cases where the disease lasted to the seventh day; most cases had already changed so favorably on the third day that the patient could be left to himself, only a proper diet being ordered.

Of all the newly recommended and of the old nearly stereotyped anti-dysenteric remedies, as mercury, lead, opium, ipecacuanha, colombo, etc., Dr. Tiedeman made use of none since the adoption of the following treatment.

After considering the state of the intestinal canal, which, independently of dysentery, may require an emetic, a purgative, or both, *nitrate of potash* is given in solution or emulsion, in doses according to the age or the constitution of the patient or the intensity of the disease. After each dysenteric evacuation, no matter how often it occurs, an injection of cold water or even of ice-water is applied.

This treatment is to be continued until tenesmus ceases, and regular evacuations will generally follow, which, in the majority of cases, happens in from six to twelve hours; when the evacuations do not take place, a dose of castor oil is administered.

When tenesmus is overcome, when the dysenteric discharges have ceased, when the fever is subdued, and fæcal evacuation is re-established, (all of which is often accomplished in twelve hours,) a solution of quinine is given and a better diet allowed.

Dr. Tiedeman says:—"In the first two stages of the disease, for every age, both sexes, every constitution, to persons of every mode of living or occupation, in every epidemic, every season, every so-called species of the disease, this treatment has proved applicable, and has often been crowned with surprising results."

The author is further of opinion that this treatment may be also useful in tropic, camp, and other so-called dysenteries, with which he is acquainted only from report.

---

*Intussusception of the Bowels.* By J. M. HAMMER, M.D., Sevierville, Tenn.

Dr. Hammer reports the following extraordinary case:—"The patient was attacked in December with acute lancinating pain in the abdomen, paroxysmal, recurring after short intervals; the pulse 90, strong and full; nausea; great anxiety. He was bled freely, anodynes were administered, but relief was not obtained for forty-eight hours; he then slowly recovered. Further details of the history and treatment are not given. A little over a month afterward, Dr. Hammer was summoned in haste to the patient, who lived at a distance of six miles. When he arrived he found the patient comfortable, but was informed that a very strange worm had been passed from the bowels. On examination the worm proved to be 'about twenty inches of the *ileum,* with its mesenteric glands,'" etc. The reporter concludes that there had been an intussusception, followed by adhesion and sloughing away of the invaginated portion of the intestines.

The health of the patient remained good until the following spring, when, after eating a hearty meal of onions and working over his farm, he became suddenly ill and died in a few hours. A post-mortem examination was not made. (*Southern Journal of the Medical and Physical Sciences,* Sept., p. 181.)

---

*Accidental Swallowing of the New Cent and other Solid Bodies.*

At a meeting of the Buffalo Medical Association, Dr. Nichols reported two cases in which the new cent, composed of nickel and copper, (coinage of 1857,) had been swallowed and passed. No unpleasant symptoms were produced in either case. In one case the coin was slightly corroded, and in the other scarcely tarnished. The less amount of copper in the new cent than in that of the old coinage, and the fact that nickel, when alloyed with other metals, seems to render the latter less liable to oxidization, renders the accident harmless, in opposition to statements made in some newspapers, in consequence of which the accident is likely to occasion needless alarm. The symptoms imputed to poisoning from this source were probably due to the use of emetics and cathartics, which are quite unnecessary.

Professor Hamilton reported, at the same meeting, a series of cases falling under his notice, in which various metallic substances had accidentally been swallowed. The most remarkable of these cases are the following:—

A gentleman, aged thirty years, swallowed a twenty dollar gold piece. Two years afterward it had occasioned no inconvenience, and he was not aware that it had ever passed.

A child, eight years old, swallowed a glass door-nob, weighing 162 grains. It passed on the third day; it occasioned only slight inconvenience.—(*Buffalo Medical Journal*, October, p. 288.)

---

*Perforation of the Cœcum and Appendix Vermiformis.*

A case of perforation of the cæcum, accompanied by impaction of fæces in the appendix vermiformis, is reported by WM. WESLEY WILKINS, M.D., in the *New Hampshire Journal of Medicine*, October, p. 289.

The patient was a boy, fifteen years of age, who came under the care of Dr. Wilkins, having been ill for a week. He was attacked at first with chills, followed by diarrhœa and vomiting, and pain in the abdomen. Death took place eight days afterwards, with the symptoms of peritonitis. The appendix was partially adherent to the cæcum, and contained a solid lump of hardened fæces half an inch in length and three-eighths of an inch in diameter. The cæcum in contact with the appendix had sloughed, and through this opening the contents of the intestine had escaped into the peritoneal sac. The evidences of peritonitis coexisted.

At the session of the New York Pathological Society in July, Dr. Sands presented a specimen illustrating ulcerative disease of the vermiform appendix, accompanied by a history of the case. The patient, an artist, aged twenty-two, while engaged in his studio, was suddenly overcome with a sense of exhaustion and fainted. He soon recovered, breakfasted, and continued at his labor till noon, when he dined freely on beefsteak and porter. Several hours afterwards he was attacked with severe pain in the right iliac region, and great prostration. Symptoms of peritonitis were declared, and he died on the fourth day after the attack. The appendix was fastened to the inferior and posterior part of the cæcum by adhesion. It was dilated about its middle from the presence of an intestinal concretion, in which were found a number of strawberry seeds, and small solid particles supposed to be earthy phosphates. The mucous membrane of the appendix was the seat of sloughing ulceration, which near its extremity had perforated the several coats, permitting the escape of feculent matter, and giving rise to acute peritoneal inflammation. Besides these morbid appearances there existed disease of the solitary and agminated glands of the small intestine, the glands having the aspect which they often present in typhoid fever.—(*American Med. Monthly*, October, p. 231.)

Professor Rochester reported to the Buffalo Medical Association a case of perforation of the appendix, and stated that during the last five years five cases had fallen under his observation. The patient in this case was a lad.

He was seen first two days before peritonitis was declared. At that time there existed simply a little diarrhœa. For the two days following he seemed to be but slightly ill. Peritonitis finally became developed, and death occurred on the fourth day. The appendix was firmly adherent to the rectum. Being carefully separated, and the cæcum removed, several small perforations were discovered. A hard body about the size of a common bean occupied the cavity of the appendix; it consisted of fæcal matter arranged concentrically around a nucleus of several stony particles of the size of a pin's head.

Professor Rochester thinks this cause of peritonitis to be more frequent than is generally supposed. It is probable that perforation of the appendix is sometimes overlooked in making post-mortem examinations. He finds the literature of the subject meagre; but it is treated of more fully by Rokitansky than by any other writer.—(*Buffalo Med. Journal,* October, p. 280.)

---

*Cases of Diseased Gall-bladder, etc.* By WILLIAM PEPPER, M.D., one of the Physicians to the Pennsylvania Hospital.

Dr. Pepper notices some of the most prominent difficulties attendant upon an investigation of the various diseases of the gall-bladder, and reports several cases with the view of inducing other observers to pay more attention to this obscure class of affections.

In case No. 1 the patient, a young man, aged twenty-six, presented a fluctuating tumor, extending nearly down to the crista of the ilium. After a preliminary exploration, a small trocar was introduced, and two or three ounces of thick muco-puriform matter, tinged with bile, escaped. A larger opening being necessary, it was resolved to employ the caustic potash for this end. The rapid decline of the patient, however, rendered it advisable to desist before the object was attained. In the mean time the tumor became tympanitic on percussion, the line of dullness varying with the position of the patient.

On inspection after death, in the right lobe of the liver were two abscesses, containing from two to three ounces of thick healthy pus, the rest of the structure of the organ being normal. The gall-bladder contained about two quarts of thick yellow puriform matter, mixed with shreds of lymph, and tinged with bile. The orifice of the cystic duct was completely occluded by layers of false membrane.

The reporter regards as probable the existence, from the commencement, of inflammation of the gall-bladder. The absence of local œdema and redness led him to doubt the existence of hepatic abscess. A cancerous aspect of the patient at one time led to the opinion that the tumor might be a medullary sarcoma of the liver. This, however, was shortly disproved by the fluctuation, and the absence of the bossilated feel which that disease generally presents.

In case No. 2 there existed cancer of the gall-bladder and pancreas. The patient was a female, aged fifty, who had been subject for several months to severe epigastric pains, with vomiting of green bilious matter. There was considerable emaciation, with the straw-colored tint characteristic of the cancerous diathesis. In the right hypochondriac region, and about midway between the umbilicus and the margin of the ribs, there was an immovable and indurated tumor, apparently about the size of the fist, and more or less tender under pressure.

On inspection after death, the gall-bladder was found to be converted into an indurated mass at least four inches long and an inch and a half thick, its increased dimensions depending upon a scirrhous degeneration of its walls. The liver was healthy. The head of the pancreas was enlarged and exceedingly firm, cutting like cartilage, but the rest of the gland appeared healthy.

In case No. 3, scirrhus of the pylorus was simulated by a gall-stone of unusual dimensions. The patient, a merchant, aged forty-five, had also chronic pleurisy affecting the right side. "About two inches to the right of the middle line, and an inch and a half below the margin of the ribs, there was an oblong tumor, deep-seated in the abdomen, but which could be readily grasped between the fingers when the abdominal muscles were relaxed; besides which there were several other indurated masses, which could be distinctly traced, owing to the attenuated condition of the abdominal parietes. These latter appeared to occupy the small intestine, and closely resembled fecal accumulations."

On inspection after death, "the tumor felt during life proved to be a large gall-stone, completely filling the bladder and moulded to its shape. Several of the convolutions of the small intestine were agglutinated, while their parietes at these points were greatly thickened and evidently in a scirrhous condition.

In case No. 4 a gall-stone obstructed the pancreatic duct, causing extensive disease of the pancreas. The patient, a married lady, aged forty-five, exceedingly obese, was seized, in October, with severe pains in the epigastric region, lasting for several days, and attended by jaundice. In a few days the pain returned with increased intensity; she became delirious, the pulse frequent, the abdomen distended and tender, and death took place in sixteen hours from the commencement of the second seizure. The liver was large and fatty; the common duct greatly enlarged, filled with bile, and its orifice obstructed by a gall-stone of the size of a small cherry, which also completely closed the pancreatic duct. The pancreas was three times its natural size; it was soft and livid, but without gangrenous odor. Peritoneum not inflamed.

In this case there was no fatty matter in the dejections during life.

In the foregoing account, the reports of the cases are greatly condensed. The remarks appended by the writer to each of the cases possess much interest. (*American Journal of the Medical Sciences*, January, p. 13.)

---

*Mercurial Muco-Enteritis.* By WM. H. BYFORD, M.D., of Evansville, Indiana.

Dr. Byford regards, as the most constant evidence of the constitutional effect of mercury, inflammation of the mucous membrane of some portion of the alimentary tube—the most frequent site of the specific inflammation being the mouth, but not always or exclusively. The irritation of the bowels so often produced before the gums are "touched," Dr. Byford is led to attribute, from careful and extended observation, to a specific mucous inflammation. The super-secretion from the liver he refers to the same condition, the function of this organ being excited just as salivation and enlargement of the salivary glands are due to the stomatitis induced by mercury. He gives two cases in illustration of these views, and sums up his conclusions in the following terms: "The specific acute inflammation produced by mercury has its site—1st, most frequently in the mouth; 2d, very frequently in the lower portion of the colon and rectum; 3d, not so often in the duodenum; 4th, situated in all these localities, it may be combined with stomatitis or not; 5th, inflammation may be produced in any of these localities either by internal use, by inunction, and probably by fumigation."—(*American Journal of the Medical Sciences*, April, p. 313.)

---

### DISEASES AFFECTING THE RESPIRATORY SYSTEM.

*Cases of Pneumonia.*—Thirteen cases of pneumonia, treated at the Bellevue Hospital, New York, are reported by GEO. S. HARDAWAY, M.D., resident-physician. The treatment consisted of dry and wet cupping; small doses of antimony in some cases; the oiled-silk jacket, and generally the pretty free use of opium, stimulants, and tonics, with a sustaining diet.

The reporter appends the following summary:—

| | | |
|---|---|---|
| "Number of cases reported | | 13 |
| " " recoveries | | 11 |
| " " deaths | | 2 |
| Per centage of mortality | | 15·4 |
| Average duration of disease until complete recovery | days | 30·2 |
| " " of those who died | " | 19 |
| " " till convalesence began | " | 16·7 |
| " " of convalescence | " | 13·4 |

"Of the two fatal cases, in one gangrene of the lung occurred, and hemorrhage; in the other instance the pneumonia was double, and accompanied by general bronchitis. Of the cases in which recovery took place, in one the pneumonia was double, and in one it was complicated with delirium tremens."—(*Nashville Journal of Medicine and Surgery*, Sept., p. 173.)

DR. NICHOLS gives the course, termination, and treatment in eleven cases received at the Buffalo Hospital of the Sisters of Charity, service of Dr. Flint. All the patients were adult males, the youngest being nineteen, and the oldest thirty-six years of age. In two cases tuberculosis was supposed to coexist, and in one the patient had lately had an attack of intermittent fever; the remaining eight cases were uncomplicated. All the cases ended in recovery, with a single exception, in which pericarditis supervened while the pneumonia appeared to be progressing favorably.

The treatment generally consisted of opium, sulphate of quinia, brandy, and nutritious diet. Bleeding, general or local, was not employed in any instance; and tartar emetic in one case only.

Three cases are given in detail, illustrating marked improvement under the use of full doses of opium. In case No. 1, opium, grs. ii. every six hours, with a little ale, was prescribed, the assemblage of symptoms being as follows:—"An aspect of anxiety and prostration; the respirations 42; dilation of alæ

nasi; pulse 120, quick and vibratory; tongue thinly coated, dry and swelled at its extremity; sordes on the teeth; expectoration tenacious, not opaque, not abundant, presenting a reddish-yellow color, pretty uniformly diffused." The whole right lung was affected. The day following, the record runs thus:—"Aspect greatly improved; pulse 104, more volume and free; respirations 26." The next day improvement was more marked: pulse 80; respirations 28, and not labored. Convalesence followed.

In case No. 2, the entire right lung was affected. The patient had been confined to the bed twelve days, and presented on his admission the following symptoms:—pulse 140; skin warm; tongue thickly coated; respirations 36. He was addicted to drinking. The treatment was, sulphate of quinia, grs. v., opium, grs. ii., every four hours; brandy, half an ounce every four hours, and sustaining diet. The day following the aspect was greatly improved. He had passed a quiet night; pulse 120; respirations 32. The next day the improvement was still more marked. The pulse was 100, and the respirations 26. Convalescence was speedily declared.

In case No. 3, pnuemonia was developed in the hospital, the patient presenting, when admitted, subacute laryngitis, and an eruption of ecthyma over the body. He was attacked with inflammation of the lower lobe of the right, and subsequently of the lower lobe of the left lung. He was treated with the sulphate of morphia, gr. $\frac{1}{4}$, at first every six, and afterward every four hours. This, with whisky, half an ounce every three hours for several days, and sustaining diet, constituted the treatment. Convalesence was established on the sixth day.—(*Buffalo Medical Journal*, Nov., p. 337.)

---

*Treatment of Pneumonia.* By C. I. POPE, M.D., of Eufaula, Alabama.

Dr. Pope attaches much importance to the use of opium in the treatment of pneumonia, bleeding, in some cases, being premised. He conjoins to the opium, calomel, and also employs the veratrum viride.—(*North-American Medico-Chirurgical Review*, July, p. 602.)

---

*On the Value of the Red Line of the Gums in the Diagnosis of Phthisis.* By D. D. SAUNDERS, M.D., and JOHN C. DRAPER, M.D., Bellevue Hospital, New York.

Dr. Theophilus Thomson, in a late work on pulmonary consumption, attaches considerable diagnostic importance to the presence of a red line at the reflected edge of the gums, especially round the incisor teeth. Drs. Saunders and Draper have undertaken to test the utility of this red line as an element in the diagnosis of phthisis. For this end, they examined all the patients under treatment in the wards of Bellevue Hospital. The results are expressed in the following tables:—

Table No. 1 is intended to show the frequency of its occurrence, without regard to the disease. The terms used at the head of the table are, *no line*, *slight*, *good*, and *excellent*. The first is used when no trace of a line exists; *slight* is used when the line is faintly marked on three or four gums; *good* is used when the line is pretty well marked on the gums of the upper jaw; *excellent* is used when the line is full, plain, and very marked on all of the gums.

The whole number of cases examined, (four hundred and fifty-one,) in whom the red line existed, is as follows :—

TABLE NO. I.

| No. of cases. | No line. | Slight. | Good. | Excellent. |
|---|---|---|---|---|
| 451 | 106 | 96 | 175 | 74 |

"Table No. 2 comprises one hundred and sixteen cases of phthisis, in all its stages. Under the head of stages, the numbers 1, 2, 3, are intended to denote the divisions commonly used in describing this disease.

TABLE NO. II.

| Stages. | No. of Cases. | No line. | Slight. | Good. | Excellent. |
|---|---|---|---|---|---|
| 1 | 26 | 7 | 3 | 9 | 7 |
| 2 | 21 | 4 | 5 | 8 | 4 |
| 3 | 69 | 17 | 17 | 21 | 14 |
| Total | 116 | 28 | 25 | 38 | 25 |

"From this we find one-fourth of the cases have no line, another fourth have it very slightly developed, leaving one-half in which it is plainly marked. It may also be observed that the stage in which the disease exists does not have any material influence on the line. Table No. 3 comprises all varieties of disease except phthisis.

TABLE NO. III.

| No. of cases. | No line. | Slight. | Good. | Excellent. |
|---|---|---|---|---|
| 335 | 78 | 71 | 137 | 49 |

"In this table we find more than three-quarters of the cases have the line more or less developed, though only a little more than one-half have it well shown. This table would rather leave the impression that this line occurs in most chronic diseases, as stated by Drs. Thompson and Frederick, oftener than in any other condition, though the following table conflicts with that opinion. Table No. 4 comprises thirty-seven cases of pregnant and recently delivered women, in whom no pathological lesion existed.

TABLE NO. IV.

| No. of cases. | No line. | Slight. | Good. | Excellent. |
|---|---|---|---|---|
| 37 | 5 | 6 | 11 | 15 |

"In the thirty-seven cases, we see the line existing in thirty-two, though only slightly in six. The results of this table deserve some consideration, as it has been stated by most authors who have investigated the subject that the line is peculiar to chronic blood diseases; this view, however, is not sustained by the above table, as no disease existed in any of the thirty-seven. It should be remarked, also, that the line occurs more frequently and is better marked in the pregnant woman than in any cases examined. The number of cases at our command will not justify a conclusion; yet they may serve as a basis of further investigation. The question might be asked—May not this line be considered in connection with the other symptoms of pregnancy?

"Table No. 5 comprises thirty-two cases of uterine disease, and is intended to show the difference between the physiological and pathological conditions of the uterus.

TABLE NO. V.

| No. of cases. | No line. | Slight. | Good. | Excellent. |
|---|---|---|---|---|
| 32 | 14 | 7 | 10 | 1 |

"In the thirty-two cases examined, there were nearly one-half where the line did not exist, and only one-third in whom it was pretty well marked. It may be observed that there is considerable difference between tables No. 4 and 5, the line occurring nearly in an increased ratio in the two conditions of the uterus, being only very well marked in a single instance when the organ is diseased.

"Table No. 6 is intended to demonstrate the influence of sex on the line.

TABLE NO. VI.

| | No. of cases. | No line. | Slight. | Good. | Excellent. |
|---|---|---|---|---|---|
| Female . . . | 234 | 57 | 55 | 80 | 42 |
| Male . . . . | 217 | 49 | 41 | 95 | 32 |

"From this we see no marked difference in the frequency of its recurrence in the different sexes.

"Table No. 7 shows the influence of age.

TABLE NO. VII.

| Age. | No. of cases. | No line. | Slight. | Good. | Excellent. |
|---|---|---|---|---|---|
| Below 20 | 48 | 21 | 9 | 10 | 8 |
| 20 to 30 | 185 | 38 | 37 | 80 | 30 |
| 30 to 60 | 199 | 42 | 44 | 78 | 35 |
| Above 60 | 19 | 5 | 6 | 7 | 1 |
| Total, | 451 | 106 | 96 | 175 | 74 |

"Age exerts no influence on the occurrence of the line.

"The following general conclusions may be drawn from the above statistics:—

"1. The red line, though it occurs frequently in phthisis and chronic blood diseases, is by no means characteristic of them.

"2. In pregnant and recently delivered women, the line occurs more frequently and better marked than in any cases examined, and may, perhaps, deserve consideration in connection with that condition.

"3. That age or sex exercise no influence on the existence of the line."—(*New York Journal of Medicine*, January, p. 62.)

---

*On the Precursory Stage of Phthisis.* By L. M. LAWSON, M.D., Professor of the Theory and Practice of Medicine and of Clinical Medicine in the Medical College of Ohio.

Professor Lawson defines the precursory stage of phthisis to be that which immediately precedes the tubercular deposit. It is "the beginning of a positive morbid action, which, if not arrested, surely and steadily progresses to the local deposit of tubercle."

He regards certain symptoms and signs as characteristic of this stage. It is marked by debility, loss of weight, impaired calorific power, variable digestion, with occasional chills and consequent febrile reaction. He thinks that in a large majority of cases passing into consumption, the pharyngo-laryngeal mucous membrane becomes early diseased, exhibiting a diffused redness, extending over the pharynx, involving more or less the uvula and soft palate, on the one hand, and dipping into the larynx, on the other. The cough and sputa are usually trivial, and are dependent, in most instances, on the throat affection. As regards a throat affection such as he describes, Professor Lawson differs from other observers, who have found its occurrence rare, except as secondary to the tubercular deposit. Hemorrhage, he thinks, may occur anterior to the deposit of tubercle, and thus, in some instances, belongs among the symptoms of the precursory stage.

The physical signs, although less marked, he regards as not less characteristic than those which supervene upon the deposit of tubercle. He enumerates the following:—Limited expansion of the chest, extending equally to both sides. Reduction of the vital capacity of the chest, denoted by the spirometer, or the common tape-measure. Diffused dullness on percussion, removed by forcible inspiration, and the parietes of the chest rigid and resisting. Weakness of the respiratory murmur, and jerking inspiration—the weakness extending to every part of the chest.

The author remarks, in conclusion, "Many will doubt the correctness of the preceding views; but my own observations have been sufficient to convince me of their truth, and that they may be readily recognized by the careful observer. All must agree that this stage is the most important in regard to therapeutics."—(*Western Lancet*, October, p. 707.)

---

*Hæmoptysis, as it prevailed in Little Rock, Arkansas, in December*, 1855, *and January and February*, 1856. By C. McAlmont, M.D.

Forty or fifty instances of hæmoptysis occurred in Little Rock during the three months above mentioned. This symptom is not of common occurrence in that situation. Tubercular affections are rarely met with, but pneumonia is frequent during the winter months.

The following is the description given by the writer:—

"The symptoms preceding and following the attack were not uniform. In some cases it came on suddenly and without previous warning, but more generally it was preceded by cold extremities and chilly sensations, loss of appetite, headache, and slight debility, with feelings of oppression. These symptoms were followed by some fever, and were either remittent or intermittent in character, lasting from one to six or eight days before the hemorrhage took place. The hemorrhage was attended and preceded by cold extremities and chilly sensations; the pulse feeble and small, beating from ninety to one hundred strokes per minute. As the hemorrhage advanced, and in proportion to the quantity of blood lost, the pulse sank and became more rapid and feeble, the body growing cold and covered with perspiration. These symptoms lasted from one to twelve hours, according to the treatment and severity of the attack. The first indications of improvement were increased strength and fullness of the pulse, followed by warmth of body and extremities, the hemorrhage growing less until it had entirely ceased, or nearly so. The reaction, in some cases, was very violent, but generally feeble. As the symptoms preceding the hemorrhage were remittent or intermittent, so was the hemorrhage with its attending symptoms; and you might look for a recurrence of the hemorrhage in one, two, or three days, if intermittent, unless cut short by treatment, and from four to six hours, if remittent, the hemorrhage never ceasing entirely until there had been several exacerbations and remissions. The hemorrhage was of a passive character, but sometimes it continued after reaction had fully taken place, and became active. This only occurred in two or three cases. The prostration following these hemorrhages was not, in general, as great as might have been expected, from the quantity of blood lost, which was great in some cases; but more often the quantity was small, ranging from two to sixteen ounces. The tongue was always more or less furred, sometimes white; more often yellow or brown. The bowels were generally costive, and showing a want of bilious secretion; the countenance *sallow;* eyes dull and surrounded with a blue circle, and, in some cases, became tinged with bile. The thirst was very great, and frequently attended with nausea and vomiting; the blood was florid and frothy; the cough attending these attacks was sometimes considerable, at other times slight, accompanied with more or less pain and tickling sensations; auscultation revealed sibilant and sonorous rattles; when the blood was in the air-passages and at points where there was dullness on percussion, (which only existed in a few cases,) there was no respiratory murmur, and in its place a crepitant rattle. * * * * * * * *

"The treatment adopted and most successful was large doses of opium and quinine, from one to three grains of the former and five to twenty of the latter, repeated in four or eight hours if necessary. Strong revulsive remedies were applied to the chest, stomach, back, and extremities; mustard plasters were generally used for this purpose; the bowels to be opened with calomel or the compound cathartic pill, the calomel to be continued in alternate doses, if indicated by want of bilious secretion and torpidity of the bowels. If the symptoms continued bad, blisters were applied to the chest and extremities. When the hemorrhage continued after reaction had fully taken place, and the pulse full and strong, venesection was resorted to, with the internal use of acid drinks, ice, and astringents. Acetate of lead is the best astringent, and was generally used, some preferring gallic acid."—(*North-Western Medical and Surgical Journal,* August, p. 343.)

---

*Paracentesis Thoracis in cases of Pleurisy.* By HENRY I. BOWDITCH, M.D.

The profession are familiar with the views and practice of Dr. Bowditch, in cases of pleurisy, from his articles on the subject in the *American Journal of Medical Sciences,* April, 1852, and in the *American Medical Monthly,* N. Y., January, 1854. The *Boston Medical and Surgical Journal,* June 4th, contains another article by Dr. Bowditch, giving the results of sixty-one operations, in thirty-seven cases, performed since the publication of his previous articles. In every instance but one, marked relief followed the operation. In the excepted case, the person was very intemperate in her habits, and was stupid with liquor when the chest was punctured; but the dyspnœa was so very great as to threaten immediate death. This patient was relieved temporarily, but sank in about twenty-four hours after the operation. In no other instance was there any untoward result. He gives the details of some (nine) of the more interesting of his recent cases.

Dr. Bowditch states that further experience has served to confirm him more than ever in "the belief of the importance of this operation as a remedial measure, to be used *not as a last resource*, but like any other simple remedy, if necessary, at any period of the disease." He sums up his experience as follows: "Since April 17th, 1850, I have operated upon sixty-two individuals, of both sexes and of all ages. I have punctured one hundred and eleven times. I know of nothing in practical medicine which has afforded me more satisfaction than this simple operation. I use designedly the expression practical *medicine*, in contra-distinction to surgery. The perfect simplicity of the operation, to one satisfied of the correctness of his diagnosis, allies it to venesection or vaccination. It is, 1st, as a general rule, less painful than a blister; 2d, it *never* does harm, if I may judge from my cases; 3d, when fluid is obtained it *always* gives relief, either temporary or permanent; 4th, very often it is the chief, if not the sole means capable of relieving severe symptoms, and even of saving life."

Dr. Bowditch adds, that he still uses the exploring trocar, although in some instances, where there has been a tendency to a re-accumulation of fluid, he has used a larger instrument.

In the *Boston Medical and Surgical Journal*, Sept. 24th, Dr. Bowditch states that he has recently resorted to paracentesis in two cases of *hydro-pneumothorax*. In both cases it had been followed by relief, and no unpleasant consequences.

---

*Lesions of the Epiglottic Cartilage.* By HORACE GREEN, M.D., L.L.D., etc.

Dr. Green has found the epiglottis to be the seat of erosions or abrasions of its mucous membrane, ulcerations of its membrane and of its glands, and œdema, much more frequently than has been supposed to be the case, exclusive of the lesions proper to phthisis as described by Louis and others. He has observed these structural alterations occurring as primary and independent affections, so far as tubercular disease is concerned; but they are complicated, in some cases, with similar lesions of the tonsils, fauces, and pharynx, and are occasionally associated with tuberculosis.

According to Dr. Green's experience, these lesions, unascertained and not suspected by the practitioner, may perpetuate indefinitely cough and expectoration, not dependent on pulmonary disease, and these symptoms are often at once relieved on discovering the morbid condition of the epiglottis and resorting to topical medication. He cites several cases in illustration of this position. To show the relative frequency of the occurrence of epiglottic lesion, he gives the following statistics:—Of four hundred and two patients affected with some form of disease of the respiratory passages, who were examined and treated between the first of May, 1856, and the 30th of April, 1857, there were found thirty-four instances of well-marked *erosions* of the epiglottis. Of this number twenty-one cases occurred in males, and thirteen in females. In upward of twenty of the above cases these lesions existed entirely independent of tubercular disease. The number of cases of ulcerations (as distinguished from *erosions*) of the epiglottis, which occurred among the four hundred and two patients, amounted to twenty-six, as estimated by Dr. Richards, the assistant of Dr. Green. About one-third of this number were females. Dr. Green, however, is of opinion that a part of these cases were more properly to be classed under the head of *erosions*. He thinks that *erosions* occur with four-fold more frequency than *ulcerations*. He states that it is difficult to give the proportion of patients affected with *œdema* of the epiglottis, for in most instances of ulceration, and in many cases of long-continued *erosion*, more or less œdema was found to be present. Dr. Richards has recorded twenty-nine cases of this lesion among the four hundred and two patients to whom reference has been made. Eight only of the twenty-nine were females.

The principal propositions embodied in the paper are summed up in the following conclusions:—

"1. The epiglottic cartilage is subject to serious alterations of structure, which, it is believed, have not received that attention in practical medicine which their importance demands. These lesions, which are ordinarily the result of inflammation, are *erosions* of the mucous membrane of the epiglottis. *Ulcerations* of the membrane and of its glands; and *œdema* or *infiltration* of its areolar tissue.

"2. Both erosions and ulcerations, although occasionally found associated with tuberculosis, yet are often found to exist as primary disease, being the antecedents, and, in many instances, the exciting cause of other grave affections.

"3. Erosions occur much more frequently than ulcerations; they differ from the latter in being more superficial, as they are confined to the mucous membrane, and ordinarily to its epithelial layer.

"4. Primary ulcerations of the epiglottis are alterations of structure, differing essentially, as we have seen, from the erosions of this organ. They originate apparently in the follicles of the mucous membrane, which soften, ulcerate, and, penetrating the fibro-cartilage, destroy, ultimately, a portion of the epiglottis, and if not arrested prove the cause of still more serious disease.

"5. Œdema of the epiglottis, or infiltration of its areolar tissue, is a lesion of this cartilage of somewhat frequent occurrence—the result, ordinarily, of catarrhal inflammation. It is attended, generally, with loss of voice, difficulty of deglutition, and is occasionally complicated with ulcerations of this cartilage, by which, in some instances, the epiglottis has been completely destroyed.

"6. The epiglottis, which is almost insensible in its normal state, becomes, when diseased, frequently the source of great irritation to the more sensitive adjacent parts. The presence of this cartilage is not indispensably necessary to insure deglutition, as deglutition may be performed in the absence of this organ. It is necessary, however, to render perfect this act; but its most important function is to cover over and protect that exquisitely sensitive portion of mucous membrane which occupies the supra-glottic space, and which is the true sentinel at the glottic opening.

"But the most important practical conclusion is found in these propositions, that some of the lesions which have now been described are often, it is believed, not only among the earliest manifestations of thoracic diseases, but are themselves, in many instances, the *true exciting cause* of these affections; and furthermore, this postulate once established, that we have it in our power, by timely topical medication, to arrest positively causes of disease which otherwise would, and in many instances do, terminate fatally."—(*American Medical Monthly*, October, p. 201.)

---

*Fistula in Ano, in its Relation to Pulmonary Tuberculosis.*
By P. H. STRONG, M.D.

Dr. Strong embodies in the following formula the doctrine generally held with regard to this subject:—"The relations of fistula in ano to pulmonary tuberculosis are such, that its presence, either as antecedent to or coexistent with the latter, is a desideratum, and to be sedulously cherished, having reference to its (pulmonary tuberculosis) prevention in the one case, and to its cure or favorable modification in the other."

Dr. Strong argues against the correctness of this doctrine, *first*, that it is based mainly on the belief of a revellent influence being exerted by the fistula, while an enlightened pathology and clinical observation teach that such an influence is of little or no value in preventing or retarding the progress of pulmonary tuberculosis; *second*, other and kindred affections, scirrhus and melanosis, depending alike on a blood dyscrasy, are not affected generally by revellent or derivant discharges; *third*, the doctrine originated when erroneous views prevailed respecting the causes, nature, and management of tubercular affections.

Conceding that tubercular matter is discharged, as it were vicariously, by means of the fistula, he contends that the proper object for therapeutics in this affection is not to eliminate the material, but to remedy the dyscrasy, by removing the conditions on which it depends.—(*Buffalo Med. Journal*, February, p. 541.)

---

*Hygienic Influences in Preventing and Arresting the Progress of Pulmonary Tuberculosis.*

Dr. Samuel A. Cartwright, of New Orleans, testifies, from his experience, to the effect of active out-door pursuits in removing the tubercular diathesis and cachexia. He attributes this effect mainly to the intimate connection between respiration and assimilation, regarding the activity of the former as the first link in the chain of influences derived from exercise. To be efficient, exercise must be involved in pursuits which interest the mind as well as occupy the muscles, and hence he remarks, "psychical influences, after all, are the most to be depended upon in the treatment and prevention of consumption."—(*New Orleans Med. and Surg. Journal*, November, p. 289.)

Dr. R. E. Haughton, M.D., of Richmond, Indiana, writing on the same subject, gives similar testimony to the value of hygienic influences, which he states to have been effectual in arresting the progress of pulmonary tuberculosis in his own person.—(*Nashville Journal of Medicine and Surgery*, May, p. 353.)

---

*Influences of Atmospherical Changes in Determining the Recurrence of Paroxysms of Asthma.*

Dr. A. W. Nichols has presented, in a tabular form, the successive paroxysms of asthma occurring in a case under observation at the Buffalo Hospital of the Sisters of Charity, in the service of Dr. Flint, from December 3d to February 17th. During this period seventeen paroxysms occurred. The date of each paroxysm; the time of the day when it took place; the degree of severity, duration, remedies employed, and the apparent effect, as well as the state of the weather, are noted in the table referred to. The paroxysms generally came on during the night or early in the morning. They did not observe any rule of periodicity. They were variable as regards intensity, and were usually much more severe after any decided change in the atmosphere. Almost all of the variations in the weather were from a cold and dry to a warmer and moister atmosphere. It is also to be noticed that no paroxysms occurred after or during certain decided atmospheric changes, as great as those which, at other times, were followed or accompanied by asthmatic attacks. The paroxysms, even when most severe, and when not influenced by remedial agents, did not continue longer than twenty hours. The inhalation of chloroform produced more marked relief than any other measure employed. The paroxysms were apparently abridged by it; the difficulty of breathing was diminished; the patient was able to assume the recumbent posture, and natural sleep soon succeeded. This was the result in six paroxysms in which it was tried. The dry bronchial râles were diminished in a few minutes after breathing chloroform.—(*Buffalo Medical Journal*, June, p. 6.)

---

*Influence of Pregnancy on the Development of Tubercles.* By EDWARD WARREN, M.D., of Edenton, North Carolina.

This subject is discussed by Dr. Warren in an essay to which was awarded the Fiske fund prize, June, 1856. The essay was published by request of the Rhode Island Medical Society. The author divides the investigation into three heads, thus:—1. A consideration of the tubercular diathesis. 2. An inquiry into the nature of tubercle. 3. An application of rules respecting disease already established. In view of the length of the essay, and the limits to which this review must be restricted, we can only present a summary of the several points of the argument by which the author endeavors to prove that pregnancy prevents the progress of phthisis even when fully developed.

"(1.) There is an inequality in the relations which men and women sustain to phthisis; the former being less liable to it than the latter.

"(2.) This inequality depends upon certain differences of conformation, etc., which are *plain, palpable, and conspicuous.*

"(3.) An examination of phthisical statistics should show that more women fall victims than men, and that the difference in the relative mortality of the two is as *plain, palpable, and conspicuous*, as their original dissimilarity of constitution and predisposition.

"(4.) An examination of statistics proves, that *it is not* a settled *fact* that more females are destroyed by this malady, and that there is a positive approximation toward *equality* in the effects of phthisis upon the two sexes.

"(5.) This 'approximation toward equality' shows the operation of some great equalizing cause, by which a certain amount of protection is secured to the female system that makes up for its greater original susceptibility, and affects the general result in the manner alluded to above.

"(6.) Pregnancy complies with *all* the conditions which this cause demands for its operation, and it is fair to attribute this protecting, preventing, and equalizing effect to its influence upon the female system."—(*American Jour. of Med. Sciences*, July, p. 87.)

---

*On the Effects of Climate on Tuberculous Disease.* By EDWIN LEE, M.R.C.S., London.

To this dissertation the Fiske fund prize was awarded, June, 1855; and it is published by request of the Rhode Island Medical Society. The author, after some preliminary remarks, considers—1st. The nature of pulmonary tuberculization. 2d. The causes of pulmonary consumption. 3d. The effect of climate on tubercular consumption; and 4th, he submits the conclusions at which he arrives. An appendix is devoted to notices of some of the places most frequented, on account of their climates, in the south of France, Italy, etc.

From the series of conclusions, which are too extended to be quoted entire, we select the points possessing most practical importance, expressing them in terms as concise as possible.

Pulmonary tuberculosis is curable in an indefinite proportion of cases, and the proportion would be increased by the more general employment of climate together with other hygienic and remedial means, now resorted to in exceptional cases only, and frequently when the disease has arrived at a stage too advanced to derive permanent amelioration from the use of any means. The formation of tubercle depends, most probably, upon an impoverishment of the blood, characterized more especially by a diminution of the normal amount of its globules, together with an alteration in its composition, occasioned chiefly by deficient activity of the skin. Statistics show that pulmonary consumption occurs more frequently in countries and localities where a humid state of the atmosphere predominates, and among classes of the population most exposed to this and other influences, which tend to depress the vital powers; and, on the other hand, tuberculous diseases are comparatively rare in cold and dry climates, and in warm and dry countries, while they are frequent in countries where the climate is moist and hot.

The chief indications in the treatment are, to remedy the morbid condition of the blood, in order to prevent or avert the morbid deposit, and to allay the general and local excitation occasioned by the organic lesion. The first of these indications is best fulfilled by change of air, and a residence, more or less prolonged, in warm countries during the winter. The beneficial influence of climate is more marked in proportion as the disease is recent. The localities best suited for the winter residence are those which, together with a suitable climate, possess resources for mental occupation and diversion, inducing patients to pass a great portion of their time in the open air. The selection of climate is to be determined by the particular circumstances in individual cases. In general, warm and dry localities best suit persons of a lymphatic or scrofulous constitution, in whom the circulation is languid; these are, on the other hand, often too exciting for individuals of a sanguine or nervous temperament, in whom there is irritability of the air-passages, a disposition to inflammation, or to hæmoptysis with acceleration of the circulation. Patients of the latter class would more frequently find themselves better where the atmosphere is somewhat moist, not liable to great transitions, and of which the action is consequently sedative. A similar climate is better adapted to patients in the more advanced stages of the disease.

Among the climates most in repute for their efficacy in retarding the progress of pulmonary consumption, there exists a considerable variety with respect to equality of temperature, the state of dryness or moisture, degree of warmth, etc. The climates of Upper Egypt and the southeastern coast of Spain are the most remarkable for their warmth and equability in winter, as well as for the dryness of their atmosphere. To these climates, Hyères, Nice, Menton, Malta, and Naples approximate nearest as respects dryness, though differing materially in other respects. The West India Islands and Cuba may be mentioned as types of hot and moist climates. Among the intermediate climates, characterized by variable degrees of warmth, equability, and humidity, are Madeira, Algiers, Pau, Pisa, Rome. The three latter have a sedative action, often depressing the vital powers of persons in health, as well as of many invalids.—(*American Journal of Med. Sciences*, April, p. 337.)

---

*Case of Pneumo-Thorax.* Reported by A. W. NICHOLS, M.D.

This case exemplifies an extremely rare form of disease, viz. the presence of air in considerable quantity within the pleural sac, the evidence of liquid effusion being entirely wanting. It occurred suddenly, during muscular exertion, the patient supposing himself to be free from any disease. It seemed most rational to attribute the occurrence to the rupture of a pleural emphysematous bleb. The details of the case are of interest with reference to the presence of air in this serous cavity for several weeks without inducing other effects than those incident to compression of the lung on the affected side; and also, as presenting the physical signs significant of the presence of air without liquid effusion in the pleural sac.—(*Buffalo Med. Journal*, April, p. 673.)

### MISCELLANEOUS.

*Report on Infant Mortality in Large Cities, the Sources of its Increase, and Means for its Diminution.* By D. MEREDITH REESE, M.D., LL.D., etc., of New York.

This report was made, by appointment, to the American Medical Association, May, 1857. It appears from the official bills of mortality that nearly one-half of the whole number of deaths, especially in large cities, occur in infancy, and under the age of five years. In the city of New York the whole mortality of the last half century amounted to 363,242, (including the still-born,) while the number of deaths under five years of age are 176,043, which is nearly 49 per cent. of the entire mortality of the city. It appears, moreover, that the infant mortality in the city of New York is fearfully on the increase. In the year 1853, the deaths under five years numbered 12,963, while in 1843 only 4588 such deaths occurred, showing the appalling increase of 8375 within ten years, which is vastly beyond the proportioned increase of the population of the city during the decennial period, as shown by the census. This increased infant mortality in 1853, as compared with 1843, is in a ratio very far beyond that of the aggregate of the deaths in persons of all ages. The deaths under five years, in 1853, were 12,963, while the deaths of all others in the city, of every age, numbered only 9739; so that the infant mortality exceeded all the other interments for that year by 3224! These facts are derived by the author from "a table of mortality in the city of New York for the fifty years between 1804 and 1853 inclusive," annexed to the report.

The statistics of Drs. Emerson and Condie, of Philadelphia, Dr. Lee, and others, show also the enormous extent of infant mortality, and its amazing increase,—the latter being a fact peculiar to our American cities, for in the great cities of the Old World the mortality of infancy has been annually diminishing for many years. The number of interments recorded as still-born, or premature births, and the increase of this number, are worthy of note. In 1843, 760 of this class were recorded, and in 1853, no less than 1930,—an increase of 1170, which is nearly 140 per cent. of increase within ten years!

In investigating the causes of this large number of cases of still-born, or premature births, the author regards it as clear that it is not to be accounted for by hereditary and constitutional vitiation of blood in either parent; contingent morbid agents, acting upon the body or mind of the mother; and incidental or accidental events occurring during pregnancy and parturition. The proportion of cases not due to these well-known causes, he thinks, may fairly be attributed to the crime of abortionism.

The dangers to life attendant upon early infancy, Dr. Reese classifies as follows:—1st. Defective vitality at birth, hereditarily transmitted from one or both parents, whereby the infant is not viable, and perishes from inanition. 2d. Mismanagement of infancy by parents, nurses, or doctors, in feeding and physicking the newly-born; depriving them of their natural nourishment, and substituting therefor the thousand slops, teas, etc., which officious grannies of both genders are wont to prepare and administer. 3d. Deficient light, pure air, ventilation, cleanliness, clothing, fuel, and wholesome food, incident especially to cities, as compared with country towns or rural districts. 4th. Illegitimate systems of practice, or quackery in its varied forms.

The means by which infant mortality is to be diminished the author considers under the following heads :—1st. Marriages should not be permitted until the parties have been subjected to professional scrutiny. Improper alliances should be prohibited by law. 2d. Foundling hospitals and lying-in asylums should be provided by the State, in order to remove the temptations to the crime of abortionism. 3d. The law should require the erection of dwellings for the poor, in accordance with the laws of health and life ; and in all cities a sanitary medical police should be established to see that this law is enforced. 4th. Hospitals for sick children should be established and properly endowed. 5th. Provisions should be made and enforced against the selling of impure or adulterated milk and other unwholesome articles of food.—(*Transactions of American Medical Association*, vol. x. 1857.)

---

*Addison's Disease of the Supra-Renal Capsules.* By DR. GOULD.

At a meeting of the Boston Society for Medical Improvement, May, 1857, Dr. Gould reported a case of the above disease, which had recently fallen under his observation at the Massachusetts General Hospital, being the first case, within his knowledge, that had been recognized in his vicinity.

The patient was a farmer, aged forty-four, who came to Boston as a passenger in an Eastern steamboat, on his way to the West, for his health. He had been a man of great energy, strength, and endurance ; rigidly temperate. Eight years before, he had pneumonia, leaving him with cough and copious expectoration, which kept him from work about three years, and at the end of this time he had a series of boils at the epigastric region, and also a large abscess in the thyroid region, which for two weeks filled and discharged every forty-eight hours. For the next four years he had excellent health. During the last year his health had been failing. He had loss of appetite, flesh, and strength, with frequent attacks of nausea and vomiting ; pain in the abdomen on stooping, with an irregular state of the bowels ; coldness, rigidity, and insensibility in the fingers of both hands, lasting perhaps an hour ; dizziness, listlessness, and loss of memory. Nothing unusual was noticed in the urinary function. A change in the complexion had existed for eight months. It was described as of a bluish cast, and at times almost purple. For several weeks discoloration within the mouth had been observed.

During the voyage to Boston he was very sea-sick, but had walked about the cabin in the morning. He was afterward found lying insensible in his berth, and was brought to the hospital in that condition. His appearance was that of a stout, vigorous man. He lay on his back quite insensible, breathing at irregular intervals ; hiccoughing, unable to swallow ; eyelids firmly closed, and the pupils contracted ; action of heart rapid and feeble ; pulse weak, uncertain, and sometimes imperceptible ; hands cold, and fingers rigidly flexed. The skin had a peculiar, dark hue, so that one person supposed that he might have had a mixture of negro and Indian blood, while another suggested his resemblance to an unwashed collier or stoker. Within the mouth, bordering the lips, was a circle of dark spots, not livid, but apparently old discolorations. The cheeks were especially dark and lustrous, and the term *bronzed* would aptly designate their appearance. The features sufficiently indicated that the peculiar hue was not due to cold; and its presence on the feet and under the clothes showed that it was not due to exposure. He died six hours after his admission.

*Sectio Cadaveris.* By DR. C. ELLIS.—"In the substance of the brain, between the lateral ventricle and longitudinal fissure, was a firm, bluish-white nodule, containing considerable cretaceous matter. On microscopic examination it was found to consist of fibroid tissue. The pleural surfaces of both sides were everywhere united by old, white, delicate fibrous tissue. The lungs crepitated throughout; in many parts were dark-red spots like ecchymoses. The heart was rather flaccid, containing a large quantity of fluid blood, but no coagula, and there was no coagulation after its removal from the body. The liver was firmly adherent to the diaphragm; its substance normal, and dark-red throughout. The spleen adhered to the diaphragm, and was rather soft. The supra-renal capsules were surrounded by fat, which was separated with difficulty from their external surface. That of the left side was much thicker than usual, very firm, and of a bluish-white color, though much of it was occupied by soft, yellow deposits, resembling tubercular matter or concrete pus. The deposit consisted of minute globules or granular corpuscles, varying in size from those of tubercle to those of pus. The firm bluish-white substance consisted of fibrous tissue. The right capsule was smaller than usual, though thicker at one part, and nowhere flat, as in the normal state. Its appearance, on section, was the same as in the other capsule, but the disease was much less extensive. The other organs were normal.

"Of the symptoms mentioned by Addison, as characteristic of the disease, we have above, from independent and unprejudiced sources, nearly every one. We have bronzed skin, debility with moderate emaciation, anemia, dyspepsia, feeble pulse, nervous twitchings, with numbness, and failure of memory. The other symptoms mentioned by him relate to the tongue, lumbar pain, the urine, (which, so far as known, was normal,) change of temper, and a peculiar odor. With regard to these, the reporters are silent, but not contradictory."—(*Boston Med. and Surg. Jour.*, July 16, p. 480.)

---

*Case of Cirrhosis of the Liver, and Bronzed Skin.* By CHARLES FRICKE, M.D., of Baltimore.

An interesting case, entitled as above, is contained in this (Medico-Chururgical) Journal, number for July, p. 604.*

---

* Analytical notices of the following articles would claim an introduction here, had the articles not appeared in the pages of this Journal. "The Psychology of Acute Rheumatism." By Dr. A. J. WHEELOCK.—(*North American Medico-Chururgical Review*, Nov., p. 922.) "On the Influences of Electrical Fluctuations as a Cause of Disease." By S. LITTELL, M.D., Surgeon to Wills Hospital for the Diseases of the Eye and Limb, Philadelphia.—(*Ibid.*, May, p. 390.)

